# Changes in Functional and Health-Related Outcomes After a Disability-Delay Program Among Community-Dwelling Older Adults

**DOI:** 10.3390/healthcare14203512

**Published:** 2026-10-19

**Authors:** Yu-Chung Chen, Yi-Chun Li, Rong-Bin Hong, Yi-Chen Shih, Jer-Hao Chang

**Affiliations:** 1 Department of Physical Medicine and Rehabilitation, Chi Mei Hospital, Liouying, Tainan 736402, Taiwan; 850616@chimei.org.tw (Y.-C.C.); 890701@mail.chimei.org.tw (R.-B.H.);; 2 Department of Occupational Therapy, College of Medicine, I-Shou University, Kaohsiung 824005, Taiwan; yichunli@isu.edu.tw; 3 Institute of Allied Health Sciences, College of Medicine, National Cheng Kung University, Tainan 701401, Taiwan; 4 Department of Occupational Therapy, College of Medicine, National Cheng Kung University, Tainan 701401, Taiwan

**Keywords:** community-dwelling older adults, occupational therapy, multidomain intervention, functional independence, activities of daily living

## Abstract

**Highlights:**

**What are the main findings?**

- Pre–post differences were observed in activities of daily living, frailty, sarcopenia risk, cognitive function, grip strength, and health-related quality of life among community-dwelling older adults participating in a 12-week occupational therapist-led multidomain community program.
- Changes in frailty, sarcopenia risk, and cognitive function showed consistent associations with concurrent changes in selected functional outcomes across separate age-adjusted regression models.

**What are the implications of the main findings?**

- Multidomain assessment and intervention planning addressing frailty, cognition, and physical function may be relevant to supporting functional independence among community-dwelling older adults.
- These findings may inform occupational therapy practice and the development of community-based programs aimed at supporting healthy aging, functional independence, and participation in daily life.

**Abstract:**

**Background:** Frailty, sarcopenia, and cognitive decline are associated with reduced functional independence and participation among older adults. Occupational therapy uses multidomain and participation-focused approaches to support functional independence and healthy aging; however, evidence regarding functional and health-related changes among community-dwelling older adults participating in structured multidomain programs remains limited. This study aimed to describe pre–post changes in functional and health-related outcomes and to explore associations among concurrent changes in these measures. **Methods:** This uncontrolled retrospective pre–post program evaluation analyzed routinely collected data from 60 community-dwelling older adults who participated in a 12-week occupational therapist-led multidomain community program at five community sites. The program was delivered once weekly for 2 h and incorporated cognitive, physical, and daily activity-related components. Functional ability, frailty, sarcopenia risk, grip strength, cognitive function, and health-related quality of life were assessed at baseline and program completion. Pre–post differences were examined using paired-samples *t*-tests, and separate age-adjusted linear regression models were used to examine associations between individual clinical change variables and concurrent changes in functional outcomes. **Results:** Pre–post differences were observed in activities of daily living, frailty, sarcopenia risk, grip strength, cognitive function, and health-related quality of life. In separate exploratory age-adjusted regression models, changes in frailty (SOF), sarcopenia risk (SARC-F), and cognitive function (SPMSQ) were associated with concurrent changes in selected functional outcomes. These associations were considered exploratory and should not be interpreted as evidence of independent prediction or causality. **Conclusions:** Pre–post changes in multiple functional and health-related outcomes were observed among community-dwelling older adults participating in the 12-week occupational therapist-led multidomain program. Given the uncontrolled retrospective pre–post design, the observed changes cannot be attributed to the program, and the associations among concurrent changes should not be interpreted as causal. These findings may inform occupational therapy practice and the development of community-based multidomain programs aimed at supporting functional independence and participation in daily life. Further prospective controlled studies are needed to confirm these findings and determine whether such programs produce clinically meaningful changes in functional and health-related outcomes.

## 1. Introduction

Dementia, frailty, and sarcopenia are common health and disability issues among older adults in the community. Dementia is a common syndrome in older adults, characterized by a decline in cognitive abilities that affects a person’s ability to live a normal life [1]. Frailty is associated with long-term adverse health consequences, including an increased risk of geriatric syndromes, dependence, disability, hospitalization, institutional placement, and death [2]. Sarcopenia is a loss of muscle function that occurs with age and is a major component of frailty. Decreased muscle mass and function are associated with poor prognosis in acute illnesses such as fragility fractures. The prevalence of sarcopenia increases with age [3]. As age increases within the older adult population, the prevalence of disability shows an upward trend. A study in Taiwan found that the prevalence rate of disability among people over 65 years old was 12.7%, and it was as high as 41.2% among those over 85 years old [4]. The gradual decline in cognitive function from mild cognitive impairment (MCI) to dementia significantly affects older adults’ ability to perform activities of daily living and their social participation. Moreover, individuals with MCI could develop severe cognitive impairment or dementia over time. According to the latest statistics from the World Health Organization, as of 2021, 57 million people worldwide suffer from dementia, and nearly 10 million new cases are reported each year [5]. This causes immense suffering for patients and caregivers, and places a heavy financial burden on families and the healthcare system [6,7]. In addition, frailty is a geriatric syndrome characterized by increased vulnerability to external stressors, closely associated with adverse outcomes including mortality, nursing home admission, and falls. Features of frailty include unintentional weight loss, fatigue, decreased physical activity, slowed walking speed, and reduced grip strength [8]. Sarcopenia is a progressive, systemic skeletal muscle disorder that typically occurs with advancing age and is associated with an increased risk of various adverse outcomes, including impaired mobility, incidence of disease, and mortality. Especially among community-dwelling older adults, it is important to understand and identify the causes of sarcopenia to prevent and intervene to improve [9,10].

From 2018 to 2026, Taiwan will transition from an aging society to a super-aged society, with the population aged 65 and above accounting for 14% to 20% of the total population [11]. During the aging process, the physiological condition of older adults gradually deteriorates, physical activity decreases, and muscle mass declines, which can lead to various subsequent diseases. Multiple chronic diseases can result in physical impairments, risks of dementia, and functional degradation, and even increasing the chance of death. In addition, as overall conditions decrease, including general weakness, sarcopenia, cognitive deterioration, and dependent daily living functions, they can lead to a decline in quality of life and may result in frequent hospitalizations, also increasing the burden on families and society.

In recent years, disability prevention and delay have become important policy goals in Taiwan. In 2017, the government introduced the “Long-Term Care 2.0 Ten-Year Plan,” which includes the “Prevention and Delay of Disability Care Plan” [12]. The policy is intended to provide community-based services for older adults with frailty, disability, or cognitive impairment. The 12-week community program examined in the present study was implemented within this policy context and included occupation-based activities addressing activities of daily living (ADLs and IADLs), cognitive training, task-oriented physical and functional activities, health self-management, environmental safety and fall prevention, and social participation. The program was delivered primarily in a group format, with individual guidance from an occupational therapist for participants who had difficulty following instructions. The policy name and intended preventive purpose are described here as contextual information and should not be interpreted as evidence that the present study established prevention or delay of disability.

Overall, much of the existing literature on aging and disability has focused on pathology and functional deficits, emphasizing disease progression and functional decline while paying relatively less attention to the remaining abilities, resources, and adaptive capacities of older adults. In contrast, the profession of occupational therapy emphasizes a strengths-based, participation-oriented perspective, focusing on individuals’ remaining functional abilities and their engagement in meaningful activities in real-life contexts. This perspective highlights the importance of supporting functional independence and social participation as key components of healthy aging [13,14,15].

From the perspective of occupational therapy, maintaining participation in meaningful daily activities is a central component of healthy aging. Occupational therapy emphasizes client-centered and occupation-based approaches that consider individuals’ remaining abilities, adaptive strategies, meaningful occupations, and environmental contexts. Community-based multidomain programs that address daily activities, cognition, physical function, and environmental adaptation may therefore be relevant to supporting functional independence and social participation among older adults [13,16,17]. Whether such programs prevent or delay subsequent functional decline or disability requires prospective controlled evaluation. However, changes in the program’s outcome measures could still offer heuristic insights.

The aim of this uncontrolled retrospective pre–post program evaluation was to describe changes in functional ability, health-related quality of life, frailty, sarcopenia risk, grip strength, and cognitive function among community-dwelling older adults who participated in a 12-week multidomain program and to explore associations among concurrent changes in these measures.

## 2. Methods

### 2.1. Participants and Study Design

This retrospective study analyzed data that had been routinely collected during the implementation of a community-based care program between 1 March 2021 and 31 December 2022. The community program had been completed before the present research was initiated. The dataset was processed in accordance with ethical guidelines and regulations, ensuring that patient identities cannot be traced. Ethical approval was subsequently obtained from the Institutional Review Board of Chi Mei Medical Center (IRB Serial No. 11206-L01; approved on 19 June 2023), with a waiver of informed consent, prior to accessing and analyzing the existing data for research purposes. This study was a retrospective analysis of pre-existing data obtained from a previously implemented community-based care program. Participants were not prospectively recruited or assigned to the intervention for the purposes of the present research, and no additional participant recruitment, intervention, or data collection was conducted specifically for this study. Formal research-oriented monitoring of treatment fidelity, detailed session-by-session progression, adherence, adverse events, and co-interventions was not prospectively established for the present retrospective analysis. Information was reported only where it could be verified from routine program records. All procedures involving human participants were performed in compliance with the ethical standards outlined in the Declaration of Helsinki. The analytical dataset included records from 60 older adults who had participated in the community program at five sites in southern Taiwan. These individuals had been enrolled in the community program before the present research analysis was initiated. There were no control groups or blind settings in this study. The program module course was designed from an occupational therapy perspective and incorporated multidomain interventions, including cognition, physical fitness, cardiorespiratory endurance, muscle endurance, and activity-based training for daily living tasks. The program details were approved by the Taiwan Ministry of Health and Welfare and covered occupation-based interventions to address individual needs and modifications. Especially, if a participant was unable to follow instructions, the occupational therapist provided individual guidance. Occupational therapists conducted a multidomain intervention program once a week for 12 weeks, totaling 12 sessions, each lasting two hours. These components aimed to support older adults’ participation in meaningful daily activities and maintain functional independence in community living.

**Figure 1:**
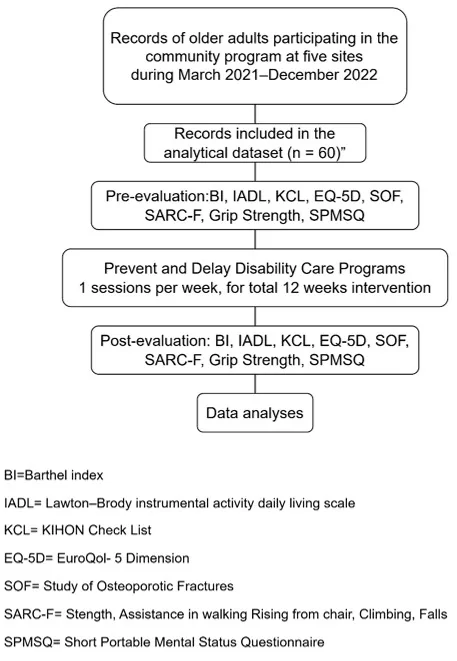
Flow diagram of retrospective record identification, assessment, and inclusion in the analysis.

Baseline and post-program assessments were available for participants included in the analytical dataset. Demographic data, including age and sex, were recorded. Functional ability was assessed using the Barthel Index (BI) and the Lawton–Brody Instrumental Activities of Daily Living (IADL) scale; frailty status using the Kihon Checklist (KCL) and the Study of Osteoporotic Fractures (SOF) index; sarcopenia risk using the SARC-F questionnaire; muscle strength using grip strength; cognitive function using the Short Portable Mental Status Questionnaire (SPMSQ); and health-related quality of life using the Taiwanese version of the EuroQol-5 Dimension (EQ-5D). Each participant underwent an initial assessment during the first week of the program and a final assessment during the last week, with the available measures collected during the same assessment session for each participant (Figure 1). Baseline and post-program assessments were compared to describe changes observed during the program period across functional ability, frailty, sarcopenia risk, muscle strength, cognitive function, and health-related quality of life. These comparisons were intended to characterize pre–post changes and were not interpreted as estimates of a causal program effect.

Because this was a retrospective analysis of routinely collected program data, several variables relevant to participant selection and intervention implementation were not systematically available. Specifically, the available records did not permit complete reconstruction of the original number of individuals screened, detailed reasons for exclusion, or the extent of incomplete records before creation of the analytical dataset. Detailed baseline diagnoses and comorbidities, individual-level session attendance and adherence, and standardized session-by-session progression of intervention intensity or task difficulty were also not systematically documented. Accordingly, these variables could not be reported or incorporated into the present analyses.

### 2.2. Measurement Tools

The Barthel Index (BI) is a commonly used assessment tool for evaluating a patient’s ability to perform daily activities and self-care. This index includes 10 basic life skills, such as eating, bathing, and dressing, and scores are assigned based on the patient’s level of independence in these activities, thereby assessing their ability to live independently. The Barthel Index ranges from 0 (completely dependent) to 100 (completely independent), helping healthcare professionals evaluate a patient’s recovery progress and develop appropriate treatment plans. This measurement was shown to have good structural validity (Kaiser–Meyer–Olkin measure of 0.904 and Bartlett’s test of sphericity *p* < 0.0001) and good inter-rater reliability (coefficients between 0.81 and 1.00) [18].

The Lawton–Brody Instrumental Activities of Daily Living (IADL) scale assigns higher scores to better performance. The Lawton–Brody IADL measures a broader set of IADLs in eight items: making telephone calls, shopping, preparing food, doing housework, doing laundry, using transportation, using pharmaceuticals, and conducting financial activities. For women, the overall IADL score ranges from 0 (low-functioning) to 8 (high-functioning). For men, the overall score ranges from 0 to 5. The inter-rater reliability of the IADL was 0.85, and its correlations with other measures of functional status ranged from 0.40 to 0.61 [19]. Because the original IADL scoring ranges differed by sex (0–5 for men and 0–8 for women), raw IADL scores were normalized by dividing each participant’s score by the corresponding sex-specific maximum score. The resulting normalized IADL score ranged from 0 to 1, with higher values indicating greater independence in instrumental activities of daily living.

The Kihon Checklist (KCL) is a screening tool to assess frailty. This assessment tool includes multiple items aimed at assessing a person’s physical function, mental status, and social interaction to help determine whether the individual exhibits signs of frailty. The purpose of the KCL is to detect signs of frailty at the early stage, allowing for appropriate intervention. Questions 1 to 5 of the Kihon Checklist focus on the training aspect of life function reconstruction. Questions 6 to 10 are designed to strengthen muscles. Questions 11 to 12 focus on dietary intake. Questions 13 to 15 are about oral function. Questions 16 to 17 are about social participation. Questions 18 to 20 concern cognitive function. Questions 21 to 25 concern psychological functioning. The higher the total score, the lower the independent life function. The KCL items 1–25 showed good validity [20].

The Taiwan version of the EuroQol-5 Dimension (EQ-5D) measures patients’ perceptions in five categories: ambulation, self-care, daily activity performance, levels of pain and discomfort, and anxiety and depression, to represent quality of life (QOL) related to health. Each category is scored from 1 to 3, in order from highest to lowest QOL. It is reported that the Taiwan EQ-5D instrument is an effective and reliable tool for measuring the health status of the general population in Taiwan. By completing this questionnaire, a comprehensive index of health-related quality of life can be obtained, which helps evaluate and compare the health status of different populations. For the present analysis, responses to the five EQ-5D-3L dimensions were summed to obtain a descriptive severity score ranging from 5 to 15, with lower scores indicating fewer reported health problems [21,22,23].

The SOF (Study of Osteoporotic Fractures) index is a self-assessment tool for frailty and comprises three indicators: (1) weight loss, (2) reduced lower limb function, which is the inability to rise from a chair five times without using the arms, and (3) reduced energy level. If one indicator is met, it is considered pre-frailty; if two or more indicators are met, it is defined as frailty. Scores range from 0 to 3, with higher scores indicating more severe frailty. In 2017, as part of the Long-Term Care 2.0 policy, the Ministry of Health and Welfare incorporated the SOF frailty assessment into the Long-Term Care 2.0 care management assessment form to prevent and delay aging-related disability. It serves as a tool for assessing frailty in community-dwelling older adults. The SOF is a valid, reliable, and independent predictor of adverse outcomes in community-dwelling older people [24,25].

SARC-F was developed by Malmstrom and Morley and used to identify whether a case is at risk of sarcopenia. It examines five areas: muscle strength, whether assistance is needed for walking, standing up from a chair, climbing stairs, and falling. A score ≥4 indicates a risk of sarcopenia (maximum score: 10). Evaluating these items can help healthcare professionals quickly assess patients’ risk of sarcopenia and implement appropriate preventive strategies. The SARC-F had good to excellent inter-rater reliability, moderate to good test–retest reliability, low to high internal consistency, low to moderate sensitivity, and moderate to high specificity [26,27,28].

Testing grip strength is a popular assessment. It is fast, easy to perform, reliable, and it produces a result that is simple to record. The test–retest reliability of the Jamar dynamometer was excellent (ICC: Jamar = 0.98, *p* < 0.001). Handgrip strength is used to assess overall strength and is associated with mortality and disability. Almost all studies on muscle strength, function, and sarcopenia in East and Southeast Asia have used handgrip strength as an indicator of skeletal muscle strength. Therefore, the Asian Working Group for Sarcopenia (AWGS) 2019 recommended using handgrip strength as a reference. Based on data from 8 Asian cohorts comprising 21,984 participants aged 65 years and older, the AWGS 2019 recommends the following diagnostic cutoffs for low muscle strength: handgrip strength <28.0 kg for men and <18.0 kg for women. Grip strength was measured using a Jamar^®^ handheld grip dynamometer, made and distributed by Performance Health Company, Warrenville, IL, US, in kilograms. Subjects were seated with shoulders adducted and elbows flexed at 90°. The dominant hand was tested twice, and the maximum value was recorded [29,30,31,32].

The Short Portable Mental Status Questionnaire (SPMSQ) is a tool used to assess cognitive function and mental status. This assessment tool includes several questions that cover memory, orientation, attention, and calculation abilities and is used to evaluate an individual’s cognitive ability and mental status. The SPMSQ is typically used to assess older adults or those at risk of cognitive impairment. A score of 0–2 errors indicates intact mental function; 3–4 errors indicate mild cognitive impairment; 5–7 errors indicate moderate cognitive impairment; and 8–10 errors indicate severe cognitive impairment. If an older adult answers three or more questions incorrectly, they need to go to the hospital for further evaluation of dementia. The intraclass correlation coefficient (ICC) for the SPMSQ was 0.89. In a test–retest reliability study, the SPMSQ demonstrated reliability in monitoring cognitive function in people with dementia. The SPMSQ consists of 10 items assessing orientation, memory, attention, and calculation. Although the conventional SPMSQ may be reported according to the number of errors, in the present dataset, cognitive performance was recorded as the sum of correct answers, ranging from 0 to 10. Therefore, higher scores indicate better cognitive performance. This scoring direction was used consistently in all analyses [33].

### 2.3. Statistical Analysis

The statistical analysis was conducted using SAS^®^ 9.4 software (SAS Institute Inc., Cary, NC, USA). Descriptive statistics were used to summarize participant characteristics and study outcomes. Pre–post comparisons were conducted using paired-samples *t*-tests for participants with available baseline and post-program measurements. For each comparison, the mean paired change, 95% confidence interval (CI) of the mean change, *p*-value, and Cohen’s dz effect size are reported in Table 1. Cohen’s dz was calculated as the mean paired change divided by the standard deviation of the paired differences. Distributional assumptions for parametric paired analyses were considered in relation to the paired change scores; because several outcomes were bounded or ordinal and the sample was modest, these analyses were treated as exploratory. The direction of change was interpreted according to the scoring convention of each instrument. Statistical significance was set at 0.05. Because multiple outcomes were examined and no single primary outcome was prospectively specified for this retrospective program evaluation, no formal multiplicity adjustment was applied; *p*-values and effect sizes should therefore be interpreted cautiously and in conjunction with the magnitude and direction of the observed changes.

Separate exploratory age-adjusted linear regression models were used to examine associations between individual clinical change variables and concurrent changes in functional outcomes. For each model, one clinical change variable was entered together with age as an adjustment covariate; no model simultaneously included all clinical explanatory variables. The dependent variables were changes in BI, IADL, KCL, or EQ-5D, as applicable. For each model, the unstandardized regression coefficient (B), 95% confidence interval (CI), *p*-value for the clinical change variable, and model-level R^2^ were reported. The R^2^ value represents the coefficient of determination for the complete age-adjusted model and was not interpreted as the independent contribution of the individual clinical change variable. Because the explanatory and outcome variables represented changes measured over the same period, temporal precedence could not be established; the analyses were interpreted as exploratory concurrent associations rather than independent prediction or causality.

## 3. Results

The analytical dataset included records from 60 community-dwelling older adults with baseline and post-program assessments. The mean age was 76.2 years; 14 participants (23.33%) were male, and 46 (76.67%) were female. Pre–post differences were observed across the assessed functional and health-related outcomes, including BI, IADL, KCL, EQ-5D, SOF, SARC-F, grip strength, and SPMSQ scores, as summarized in Table 1.

Changes in Functional Ability: Significant pre–post differences were observed in measures of functional ability. Barthel Index (BI) and Lawton–Brody Instrumental Activities of Daily Living (IADL) scores were significantly higher at the post-program assessment than at baseline.

Changes in Frailty Status: Pre–post differences were observed in frailty-related measures, with lower Kihon Checklist (KCL) and Study of Osteoporotic Fractures (SOF) index scores at the post-program assessment than at baseline.

Changes in Sarcopenia-related Measures: Significant pre–post differences were observed in sarcopenia-related measures. Grip strength was significantly higher at the post-program assessment than at baseline, whereas SARC-F scores were significantly lower.

Cognitive Function and Quality of Life: Significant pre–post differences were observed in SPMSQ and EQ-5D scores. SPMSQ scores were significantly higher at the post-program assessment than at baseline, whereas EQ-5D descriptive severity scores were significantly lower.

Changes in these clinical measures were subsequently examined in separate exploratory age-adjusted regression models, with each clinical change variable entered individually together with age as an adjustment covariate, to assess its association with concurrent changes in functional outcomes.

As shown in Table 2, separate age-adjusted linear regression models were used to examine the associations between changes in SOF, SPMSQ, SARC-F, and grip strength and concurrent changes in BI. The corresponding model-level R^2^ values were 0.47, 0.37, 0.19, and 0.13, respectively. Changes in SOF (B = −7.33, 95% CI: −9.68 to −4.98, *p* < 0.01), SPMSQ (B = 3.05, 95% CI: 1.77 to 4.32, *p* < 0.01), and SARC-F (B = −2.90, 95% CI: −5.29 to −0.52, *p* = 0.02) were significantly associated with concurrent changes in BI. In contrast, the association between change in grip strength and change in BI was not statistically significant (B = −0.77, 95% CI: −2.04 to 0.49, *p* = 0.22).

As shown in Table 2, univariate regression with an age-adjusted model was used to examine the associations between changes in SARC-F, SOF, and SPMSQ and concurrent changes in IADL. The corresponding model-level R^2^ values were 0.31, 0.24, and 0.20, respectively. Changes in SARC-F (B = −0.11, *p* < 0.01), SOF (B = −0.11, *p* < 0.01), and SPMSQ (B = 0.05, *p* < 0.01) were significantly associated with concurrent changes in IADL.

In addition, univariate regression with an age-adjusted model was used to examine the associations between changes in SOF, SARC-F, SPMSQ, and grip strength and concurrent changes in KCL. The corresponding model-level R^2^ values were 0.29, 0.29, 0.27, and 0.27, respectively. Changes in SOF (B = 2.42, *p* < 0.01), SARC-F (B = 1.99, *p* < 0.01), SPMSQ (B = −1.14, *p* < 0.01), and grip strength (B = 0.97, *p* < 0.01) were significantly associated with concurrent changes in KCL.

Finally, the same model was used to examine the associations between changes in SARC-F, SOF, and SPMSQ and concurrent changes in EQ-5D. The corresponding model-level R^2^ values were 0.28, 0.26, and 0.22, respectively. Changes in SARC-F (B = 0.59, *p* < 0.01), SOF (B = 0.70, *p* < 0.01), and SPMSQ (B = −0.31, *p* < 0.01) were significantly associated with concurrent changes in EQ-5D. Therefore, changes in selected frailty-, sarcopenia-, and cognition-related measures were associated with concurrent changes in functional outcomes. These associations should be considered exploratory and should not be interpreted as independent predictive effects.

## 4. Discussion

### 4.1. Observed Pre–Post Changes in Functional and Health-Related Outcomes

As shown in Table 1, pre-post differences were observed across multiple functional and health-related indicators during program implementation. Previous research has explored multidomain community engagement approaches that combine physical, cognitive, and health-promotion elements to improve older adults’ cognitive function, physical abilities, and activity levels, thereby helping maintain their functional independence [34,35,36]. However, research results vary depending on population, intervention characteristics, and outcome measures [37,38,39,40]. The high baseline Barthel Index score in the present sample suggests a potential ceiling effect, which may have limited sensitivity to further change in basic activities of daily living. Because the present study lacked a concurrent comparison group, the observed differences cannot be attributed specifically to the program.

This study used functional and risk-related assessments in a community-dwelling older population rather than restricting the sample to people with a specific diagnosis. The program incorporated cognitive training, physical activities, and daily activity-related components. Such multidomain approaches may be relevant to supporting functional independence, healthy aging, and participation in daily life among older adults [41,42,43]. The present findings may inform the development and implementation of similar community-based programs; however, whether such programs prevent or delay functional decline or disability requires prospective controlled evaluation.

Previous studies have examined community-based interventions for people with dementia and have assessed outcomes related to cognitive function, quality of life, physical function, emotional well-being, and caregiver burden [44,45,46]. These outcome domains provide important information; however, broader consideration of functional independence and participation in daily life may provide a more comprehensive perspective on the needs of community-dwelling older adults. Separate exploratory age-adjusted regression models were used to examine associations between changes in selected clinical measures and concurrent changes in functional outcomes among community-dwelling older adults. These analyses were exploratory and were not intended to establish causal relationships or identify independent predictors. The observed associations may inform future intervention planning and the development of community-based multidomain programs, but their clinical relevance requires confirmation in prospective controlled studies.

The available retrospective records did not support classifying all participants as undiagnosed or as being at a uniform level of risk for dementia, sarcopenia, or frailty. Accordingly, the present study is best understood as an evaluation of community-dwelling older adults with varying cognitive, physical, and functional status. Early identification of functional vulnerabilities may provide useful information for multidimensional assessment and monitoring, but the data of this study design cannot determine whether intervention at an earlier stage prevents subsequent functional decline.

The observed associations among changes in KCL, SARC-F, BI, and IADL should also be interpreted in light of conceptual and measurement overlap among these instruments. KCL and SARC-F include items reflecting mobility and functional task performance, whereas BI and IADL directly assess independence in daily activities. Consequently, some of the observed concurrent associations may partly reflect shared underlying functional constructs or overlapping item content rather than relationships among entirely distinct domains.

The community-based program was designed to support older adults across varying levels of cognitive and functional status rather than exclusively targeting individuals with a specific clinical diagnosis. Screening measures including SOF, SARC-F, SPMSQ, and KCL were used to characterize frailty-, sarcopenia-, cognition-, and function-related status within the available dataset. This multidimensional approach extends beyond a disease-centered perspective by emphasizing functional independence, healthy aging, and participation in daily life; however, the screening results should not be interpreted as formal diagnoses or as evidence that the program prevented disability.

A recent single-group single-arm pre–post study also examined a long-term community-based adaptive physical activity program for older adults. At the end of an 8-month intergenerational Taekwondo adaptation program, some functional performance indicators (particularly aerobic endurance and coordination) showed changes before and after the program, while others did not show significant changes. It is noteworthy that the authors emphasize that the lack of a control group limits the causal interpretation of the observed changes. Although the interventions and outcome indicators of this study differ from ours, these findings provide contemporary comparative evidence supporting a cautious interpretation of functional changes observed in community-based physical activity programs for older adults [47].

Because this study used an uncontrolled retrospective pre–post design without a concurrent comparison group, the observed changes cannot be attributed specifically to the program. Alternative explanations, including regression to the mean, practice effects from repeated assessments, natural changes over time, concurrent healthcare or community-based services, and other unmeasured factors, cannot be excluded. Therefore, the findings should be interpreted as observed pre–post changes and concurrent associations rather than evidence of program effectiveness or causality.

### 4.2. Factors Associated with Concurrent Changes in Functional Outcomes

This study adopted separate age-adjusted linear regression models to explore associations between changes in selected clinical measures and concurrent changes in BI, IADL, KCL, and EQ-5D. These measures reflect different aspects of functional ability, frailty, and health-related quality of life among community-dwelling older adults. The analyses were intended to explore patterns of concurrent change observed during the program period rather than to identify independent predictors or establish causal relationships. Although the program was designed to support functional independence and health-related outcomes among older adults, the present uncontrolled retrospective pre–post study cannot determine whether the observed changes were attributable to the program.

As shown in Table 2, changes in SOF were significantly associated with concurrent changes in BI, IADL, KCL, and EQ-5D in separate age-adjusted models; the corresponding model-level R^2^ values ranged from 0.24 to 0.47. These R^2^ values reflect the full model, including age, and should not be interpreted as the independent contribution of SOF. Previous literature has reported associations between frailty and limitations in muscle strength, mobility, balance, activities of daily living, and health-related quality of life [46,48,49]. Related studies have also shown that the more severe the frailty, the lower the quality of life, which may be due to decreased physical function, increased psychological stress, reduced social activities, increased economic burden, and decreased independence [25,49,50]. In the present study, the associations between concurrent changes in frailty-related measures and functional outcomes do not establish that reducing frailty would improve daily functioning or health-related quality of life.

In the age-adjusted regression models, changes in SARC-F were significantly associated with concurrent changes in BI, IADL, KCL, and EQ-5D; the corresponding model-level R^2^ values ranged from 0.19 to 0.31. Sarcopenia risk and reduced muscle function have been associated with limitations in mobility and daily activities in previous research [51,52,53,54]. In the present study, changes in SARC-F scores were associated with concurrent changes in functional and health-related outcomes, but the direction and causal nature of these relationships cannot be determined from the retrospective pre–post analyses.

Changes in grip strength were significantly associated with concurrent changes in KCL in the corresponding age-adjusted model, whereas the association between change in grip strength and change in BI was not statistically significant (B = −0.77, 95% CI −2.04 to 0.49, *p* = 0.22). These findings should be interpreted as model-specific associations rather than evidence that changes in grip strength produced changes in functional outcomes.

In the regression model, changes in SPMSQ were significantly associated with concurrent changes in BI, IADL, KCL, and EQ-5D; the corresponding model-level R^2^ values ranged from 0.20 to 0.37. Lower cognitive screening scores have been associated with greater limitations in daily activities and other adverse outcomes in previous studies [50,55,56]. In the present study, changes in SPMSQ scores were associated with concurrent changes in daily living functions and health-related quality of life. These associations do not establish temporal direction, independent prediction, or causality.

The regression findings should therefore be interpreted as exploratory patterns of concurrent change. Model-level R^2^ values reflect the full age-adjusted models, not the independent contribution of individual clinical variables. In addition, the reported B coefficients are unstandardized coefficients and should not be interpreted as effects associated with a one-standard-deviation change.

Consistent with the study objectives, changes in frailty (SOF), sarcopenia risk (SARC-F), and cognitive function (SPMSQ) were associated with concurrent changes in selected functional and health-related outcomes in separate age-adjusted regression models. These findings highlight potentially relevant domains for multidimensional assessment and monitoring but should not be interpreted as evidence of independent prediction, program effectiveness, or causality.

On the other hand, the participating communities were located in relatively rural areas, where transportation barriers and the lack of caregivers to accompany older adults living alone limited their ability to attend the community module program course. These factors reduced older adults’ access to health-promoting resources.

### 4.3. Clinical Implications for Occupational Therapy Practice

From an occupational therapy perspective, the present findings highlight the relevance of multidimensional assessment of daily functioning, frailty, sarcopenia risk, muscle strength, cognition, and health-related quality of life in community-dwelling older adults. The observed pre–post differences in BI, IADL, and related measures occurred during the program period but cannot be attributed specifically to the program. Community-based programs integrating physical activity, cognitive stimulation, and daily activity training may provide a useful framework for supporting participation and functional independence, but the effectiveness of such programs still requires prospective controlled evaluation.

The observed changes in functional ability, frailty, sarcopenia risk, cognition, and health-related quality of life occurred concurrently during the program period. These domains may be relevant for occupational therapy assessment and monitoring; however, the observed associations do not establish that changes in frailty, sarcopenia risk, or cognition independently produced changes in functional outcomes. The findings should therefore be viewed as hypothesis-generating. Future prospective controlled studies should evaluate whether multidomain occupational therapy programs produce clinically meaningful and sustained changes in functional independence and participation in daily life.

### 4.4. Contribution to the Literature

Previous studies on aging and functional decline have frequently examined older adults with specific conditions, including dementia, frailty, and sarcopenia. These conditions have been associated with limitations in activities of daily living, reduced mobility, and poorer quality of life. Intervention studies involving these populations have explored strategies to maintain functional capacity, support independence in daily activities, and reduce care needs [30,31,32,33,34,35,36]. In contrast, the present study focused on community-dwelling older adults with varying levels of functional, physical, and cognitive status rather than restricting the sample to individuals with a specific diagnosis. However, focusing primarily on diagnosed populations may provide limited information about the functional needs of community-dwelling older adults with varying levels of physical, cognitive, and functional vulnerability. Functional changes may develop gradually over time, highlighting the relevance of early identification and multidimensional assessment to support functional independence, participation in daily life, and healthy aging among community-dwelling older adults.

Building on prior research, this study applied assessment tools commonly used to evaluate cognitive function, frailty, and sarcopenia risk, including the SPMSQ, SOF, SARC-F, and KCL, to a broader population of community-dwelling older adults. This multidimensional assessment approach enabled the concurrent examination of functional, physical, and cognitive characteristics and provided a framework for describing changes observed during participation in a community-based multidomain program. By extending the focus beyond disease-specific populations, this study contributes to understanding the functional and health-related characteristics of community-dwelling older adults and may inform the development of community-based strategies to support functional independence, participation in daily life, and healthy aging.

## 5. Limitations

This study has several limitations inherent to its uncontrolled retrospective pre–post design. Without a concurrent comparison group, the observed pre–post changes cannot be attributed specifically to the program. Regression to the mean, practice effects from repeated assessments, natural changes over time, concurrent healthcare or community-based services, and other measured or unmeasured confounding factors cannot be excluded. The analyses should therefore be interpreted as descriptive pre–post comparisons and exploratory associations rather than evidence of program effectiveness or causality.

The routine records also contained incomplete information on participant selection and program implementation. The original number of individuals screened, the detailed reasons for exclusion, and the extent of incomplete records prior to the creation of the analytical dataset could not be fully reconstructed. Detailed diagnoses and comorbidities, individual-level attendance and adherence, standardized session-by-session progression of intervention intensity or task difficulty, formal treatment-fidelity measures, adverse events, and concurrent interventions were not systematically documented. These limitations restrict assessment of selection bias, intervention exposure, fidelity, and potential co-intervention effects and should be considered when interpreting the findings.

The sample was drawn from five community sites in southern Taiwan and included older adults with heterogeneous functional, physical, cognitive, educational, and life-experience characteristics. Site-level clustering was not modeled, and the small number of sites limits the ability to separate individuals from site-level influences. These features, together with local contextual differences and transportation or caregiver-access barriers, limit the generalizability of the findings beyond the participating communities.

The program followed a 12-week schedule of one 2 h session per week. However, the retrospective records did not provide sufficiently detailed individual-level data on attendance, adherence, or progression to characterize actual intervention exposure. In addition, follow-up data after program completion were unavailable, so the sustainability of the observed pre–post changes could not be evaluated. Future prospective studies should incorporate standardized monitoring of fidelity and adherence, as well as longer-term follow-up assessments.

Concurrent care from other health professionals or community services was not systematically recorded and could not be controlled analytically. Therefore, any contribution of occupational therapy or other concurrent services to the observed changes cannot be isolated in the present study.

Formal regression diagnostic assessments of linearity, residual normality, homoscedasticity, and influential observations were not performed in the original analysis. Therefore, potential violations of linear regression assumptions cannot be excluded, and the exploratory regression findings should be interpreted with caution.

The program was implemented during 2021–2022, when the COVID-19 pandemic may have influenced community attendance, physical and social activity, access to health services, and participant selection. These pandemic-related influences were not systematically captured in the routine dataset and may have affected the observed pre–post changes.

Future research should employ a prospective, controlled design, increase the number of participants, clearly define eligibility procedures, provide complete baseline diagnostic and comorbidity information, standardize attendance and adherence monitoring, record concurrent interventions, and conduct longer-term follow-ups to determine whether multidomain community programs can produce clinically meaningful and sustainable changes.

## 6. Conclusions

Among 60 community-dwelling older adults included in this uncontrolled retrospective pre–post program evaluation, differences between baseline and post-program assessments were observed across multiple functional and health-related measures. Separate age-adjusted regression models identified associations among concurrent changes in selected frailty-, sarcopenia-, cognition-, and function-related measures. These findings are descriptive and exploratory and do not establish independent prediction, program effectiveness, prevention or delay of disability, or causality.

The findings may inform multidimensional occupational therapy assessment and the design of future community-based programs aimed at supporting functional independence and participation in daily life. Prospective controlled studies with standardized monitoring of participant selection, attendance, adherence, intervention fidelity, concurrent interventions, and longer-term follow-up are required to determine clinical effectiveness and sustainability.

## Figures and Tables

**Table 1:** Descriptive statistics and pre–post comparisons of functional and health-related outcomes.

	Pre	Post	Δ Change	*p*	95%CI of Δ	Cohen’s *d*_z_
	Mean ± SD					
Age (year)	76.2 ± 6.31					
Sex, *n* (%)						
Male	14 (23.33)					
Female	46 (76.67)					
BI (range, 0–100; higher is better)	91.42 ± 11.86	99.33 ± 2.68	7.92 ± 10.51	0.01	5.20 to 10.63	0.75
IADL (Normalized IADL score (range, 0–1; higher is better)						
Male	0.83 ± 0.26	0.97 ± 0.11	0.14 ± 0.20	0.02	0.03 to 0.26	0.70
Female	0.76 ± 0.30	0.92 ± 0.22	0.16 ± 0.21	<0.01	0.10 to 0.22	0.76
KCL (range, 0–25; lower is better)	6.83 ± 5.45	1.9 ± 3.03	−4.93 ± 4.26	<0.01	−6.03 to −3.83	−1.16
EQ-5D descriptive sum score (range, 5–15; lower is better)	6.17 ± 1.67	5.22 ± 0.9	−0.95 ± 1.21	<0.01	−1.26 to −0.64	−0.79
SOF (range, 0–3; lower is better)	0.7 ± 0.87	0.02 ± 0.13	−0.68 ± 0.87	<0.01	−0.91 to −0.46	−0.78
SARC-F (range, 0–10; lower is better)	1.55 ± 2.21	0.48 ± 1.74	−1.07 ± 1.07	<0.01	−1.34 to −0.79	−1.00
Grip Strength (higher is better)						
Male	29.79 ± 6.64	31.51 ± 6.86	1.73 ± 1.26	<0.01	1.00 to 2.45	1.37
Female	20.55 ± 5.06	21.73 ± 5.46	1.17 ± 2.25	<0.01	0.51 to 1.84	0.52
SPMSQ (range, 0–10; higher is better)	7.4 ± 2.16	8.77 ± 0.74	1.37 ± 1.78	<0.01	0.91 to 1.83	0.77

BI = Barthel Index, IADL = Lawton–Brody Instrumental Activities of Daily Living scale, SOF = Study of Osteoporotic Fractures, KCL = Kihon Checklist, SPMSQ = Short Portable Mental Status Questionnaire, EQ-5D = EuroQol-5 Dimension, SARC-F = Strength, Assistance in walking, Rising from chair, Climbing, Falls. Δ—the difference between the scores pre-evaluation and post-evaluation. IADL scores were normalized to a 0–1 scale by dividing each participant’s raw score by the sex-specific maximum score (5 for men and 8 for women). The 95% CIs and Cohen’s *d*_z_ values were calculated from the reported paired-change mean and SD.

**Table 2:** Separate age-adjusted linear regression models examining associations between clinical changes and concurrent changes in functional and health-related outcomes.

			95% Confidence Intervals	
Difference in Pre–Post Evaluation	Estimate (B)	*p*	Lower	Higher
ΔBI				
ΔSOF (R^2^ = 0.47)	−7.33	<0.01	−9.68	−4.98
ΔSPMSQ (R^2^ = 0.37)	3.05	<0.01	1.77	4.32
ΔSARC-F (R^2^ = 0.19)	−2.90	0.02	−5.29	−0.52
ΔGrip strength (R^2^ = 0.13)	−0.77	0.22	−2.04	0.49
ΔIADL				
ΔSARC-F (R^2^ = 0.31)	−0.11	<0.01	−0.15	−0.06
ΔSOF (R^2^ = 0.24)	−0.11	<0.01	−0.17	−0.06
ΔSPMSQ (R^2^ = 0.20)	0.05	<0.01	0.02	0.08
ΔKCL				
ΔSOF (R^2^ = 0.29)	2.42	<0.01	1.31	3.52
ΔSARC-F (R^2^ = 0.29)	1.99	<0.01	1.08	2.90
ΔSPMSQ (R^2^ = 0.27)	−1.14	<0.01	−1.69	−0.58
ΔGrip strength (R^2^ = 0.27)	0.97	<0.01	0.51	1.44
ΔEQ-5D				
ΔSARC-F (R^2^ = 0.28)	0.59	<0.01	0.33	0.85
ΔSOF (R^2^ = 0.26)	0.70	<0.01	0.38	1.02
ΔSPMSQ (R^2^ = 0.22)	−0.31	<0.01	−0.48	−0.15

BI = Barthel Index, IADL = Lawton–Brody Instrumental Activities Daily Living scale, SOF = Study of Osteoporotic Fractures, KCL = Kihon Checklist, SPMSQ = Short Portable Mental Status Questionnaire, EQ-5D = EuroQol-5 Dimension, SARC-F = Strength, Assistance in walking, Rising from chair, Climbing, Falls. Δ—the difference between the scores of pre-evaluation and post-evaluation. The *p*-value corresponds to the unstandardized coefficient for the clinical change variable in the corresponding age-adjusted model. Each row demonstrates a univariate regression with age as an adjustment covariate. B = unstandardized regression coefficient; R^2^ = coefficient of determination for the corresponding age-adjusted model.

## Data Availability

The data presented in this study are available on request from the corresponding author due to institutional and ethical restrictions concerning participant privacy and confidentiality.
